# Physical Activity during Pregnancy and Offspring Neurodevelopment and IQ in the First 4 Years of Life

**DOI:** 10.1371/journal.pone.0110050

**Published:** 2014-10-28

**Authors:** Marlos R. Domingues, Alicia Matijasevich, Aluísio J. D. Barros, Iná S. Santos, Bernardo L. Horta, Pedro C. Hallal

**Affiliations:** 1 Sports Department, Federal University of Pelotas, Pelotas, Rio Grande do Sul, Brazil; 2 Postgraduate Programme in Epidemiology, Federal University of Pelotas, Pelotas, Rio Grande do Sul, Brazil; 3 Department of Preventive Medicine, School of Medicine, University of Sao Paulo, Sao Paulo, Brazil; University of Cincinnati, United States of America

## Abstract

**Background:**

Maternal physical activity during pregnancy could alter offspring's IQ and neurodevelopment in childhood.

**Methods:**

Children belonging to a birth cohort were followed at 3, 12, 24 and 48 months of age. Physical activity during pregnancy was assessed retrospectively at birth. Neurodevelopment was evaluated by Battelle's Development Inventory (12, 24 and 48 months) and IQ by the Weschler's Intelligence Scale (48 months). Neurodevelopment was based on Battelles' (90th percentile) and also analyzed as a continuous outcome. IQ was analyzed as a continuous outcome. Potential confounders were: family income, mother's age, schooling, skin color, number of previous births and smoking; and newborns': preterm birth, sex and low birth weight.

**Results:**

From birth to 48 months, sample size decreased from 4231 to 3792. Crude analysis showed that IQ at 48 months was slightly higher (5 points) among children from active women. The Battelle's score at 12 and 24 months was higher among offspring from active mothers. After controlling for confounders, physical activity during pregnancy was positively associated to the Battelle's Inventory at 12 months IQ, however, at 48 months no association was observed.

**Conclusion:**

Physical activity during pregnancy does not seem to impair children's neurodevelopment and children from active mothers presented better performance at 12 months.

## Introduction

Physical activity during pregnancy is known to result in health benefits as much as among non-pregnant individuals. For example, women who exercise while pregnant experience less muscular discomforts and depressive symptoms, keep their weight gain within normal ranges and present lower blood pressure and blood sugar levels [1]. Evidence also shows that children may benefit from maternal exercise, as preterm birth is less frequent among exercisers [2], [3]. Besides, active mothers are more likely to present healthier lifestyles that may influence future health outcomes such as future diabetes and hypertension [1], [4], [5].

However, as it is observed in the general population, physical activity (PA) level during gestation is below the recommended by health guidelines [6], [7]. In Brazil, specifically in this population, leisure-time physical activity prevalence is very low and few women attain the current guidelines of 150 minutes per week [6]. Many reasons are reported by women to not exercise [8], and even among those who were previously active, unexplained fear of harming herself or fetus is observed [6], [7]. Although international guidelines recommend PA during pregnancy [9], [10], in many countries physical activity counseling is not routine during antenatal care.

In the 1980's, when physical activity in pregnancy became a research subject, one of the first findings was that children from exercising mothers were thinner, as they were born with less body fat [11]. Although lighter and thinner, these babies did not present low birth weight, but one of the concerns was that lower body fat could negatively affect neurodevelopment, as the nervous system depends largely on fat to develop. Future studies did not support such concern and evidence showed that children from exercising mothers perform equal or slightly better on neurodevelopment scales [11], [12], [13].

The aim of this study was to evaluate if leisure-time physical activity (LTPA) during pregnancy could alter offspring's IQ and neurodevelopment during childhood in a Brazilian birth cohort.

## Materials and Methods

A birth cohort started in 2004 in the city of Pelotas (southern Brazil), when all births (from January 1^st^ to December 31^st^) were identified and those live borns whose family lived in the urban area of the city recruited to the study. The study was hospital based (where more than 99% of births happen), but also included women delivering elsewhere, as they were referred to a hospital soon after birth. Mothers were interviewed and children were measured (perinatal interview) in the first 24 hours after delivery. Mothers were visited at home or went to the research center for future assessments when children were at the ages of 3, 12, 24 and 48 months. At the perinatal interview (hospital) we included all live births and stillbirths when birth weight was above 500 g from women living in the urban area of Pelotas. Along with maternal measurements and interviews, children were also measured and evaluated for several health outcomes, including mental health and neurodevelopment. The methods of the cohort are best described elsewhere [14], [15].

Physical activity (PA) during pregnancy was assessed retrospectively during the perinatal interview using a questionnaire (developed and tested by the research team) to collect information on activities performed during leisure time in each trimester of gestation. The following variables were used to describe PA during pregnancy: PA during pregnancy (yes/no), considering any leisure activity in any trimester; any PA during first, second or third trimester; PA during each trimester (yes/no) considering the cutoff point of 150 minutes per week; and tertiles of minutes spent in PA, with a fourth category for women that reported no activity at all. A deeper description of PA patterns in this sample was previously published [6]. Household, commuting and occupational activities were not assessed.

Child development was assessed using two instruments, the screening version of Battelle's Development Inventory [16], that indicates suspected developmental delay, and IQ that was measured with the Wechsler Preschool and Primary Scale of Intelligence (WPPSI) [17]. Battelle's Inventory was administered at the ages of 12, 24 and 48 months, while the IQ test was used at the 48 months assessment. The Battelle's Inventory is a screening tool appropriate to be used from birth to 8 years and helps to identify disabilities among children, school readiness, interaction communication skills, attention, motor skills, and memory among other characteristics of children's neurodevelopment.

The screening version of Battelle's Development Inventory provides a continuous score that was categorized based on the 90th percentile (P90 - cutoff point) of the score obtained in our sample (best development results). An alternative analysis of the Battelle's score was also performed using the score as a continuous outcome. The IQ was analyzed as a continuous variable.

Statistical analysis (Stata 11.0) described the sample (crude analysis) according to demographics, behavior, development scores and physical activity variables. Multivariable models (adjusted analysis) were used to control for potential confounders, Poisson regression was used to measure the association between Battelle's outcomes and physical activity while a linear regression was used to study the influence of LTPA on IQ scores. The variables used for analysis were: family income (quintiles), maternal schooling (four groups), smoking during pregnancy (yes/no), maternal age and skin color (white/black/mixed), maternal occupation characteristics during pregnancy (standing for long periods and lifting heavy weights at work), number of previous births, maternal depressive symptoms at 48 months (based on the Edinburgh Postnatal Depression Scale), birth weight, child's sex and preterm birth (based on an algorithm that considered last menstrual period or ultrasound scans or Dubowitz score).

The study protocol was approved at each follow-up by the Medical Research Ethics Committee of the Federal University of Pelotas Medical School, affiliated with the Brazilian Federal Medical Council. Written informed consent was obtained from all mothers before all interviews. Whenever the mother was not in charge of taking care of the child, and the interviewee was another person, such as a close relative, legal guardian or caretaker, an informed consent was signed by this person before the interview.

## Results

The first interview of the birth cohort (at hospital) included 4231 newborns. From birth to 48 months, due to losses to follow-up at 3, 12, 24 months of age, this number gradually decreased to 3792.

Table 1 describes the sample with respect to maternal and child characteristics (maternal age, schooling, skin color, physical activity during pregnancy, family income, smoking, depressive symptoms and child's sex, birth weight and preterm birth). Mothers were mostly white, in the 20–35 years age group, 27.5% smoked during pregnancy and only 13.3% of mothers reported any leisure-time physical activity (LTPA) during pregnancy. The proportion of mothers attaining the cutoff point of 150 minutes of physical activity per week decreased from 6.6% in the first trimester of gestation to 3.5% in the third. The value of PA tertiles in minutes for each trimester is shown in Table 2 along with weekly time range of physical activity in each trimester. Nearly 15% of children were born preterm and 9% presented low birth weight (<2500 g). The average child's IQ at 48 months was 99.6 (sd  = 16.7) points.

**Table 1:** Maternal and children's characteristics. Pelotas 2004 Birth Cohort (N = 4147).

	N(%)
Mother's age	
≤19	792 (19.1)
20–35	2800 (67.6)
36– +	553 (13.3)
Maternal Schooling (at birth)	
0–4	639 (15.5)
5–8	1691 (41.2)
9–11	1362 (33.2)
12– +	414 (10.1)
Skin color	
White	3030 (73.0)
Black	828 (20.0)
Mixed	289 (7.0)
Smoking during pregnancy	
Yes	1142 (27.5)
No	3005 (72.5)
Heavy lifting at work	
Yes	343 (8.3)
No	3804 (91.7)
Long standing at work	
Yes	937 (22.6)
No	3210 (77.4)
Preterm Birth	
Yes	602 (14.6)
No	3533 (85.4)
Low birth weight	
Yes	372 (9.0)
No	3772 (91.0)
Sex of the newborn	
Boys	2157 (52.0)
Girls	1990 (48.0)
Maternal Depression at 48 m (Edimburgh)	
Yes (10+)	1107 (29.5)
No (0–9)	2641 (70.5)
LTPA during pregnancy	
Yes	553 (13.3)
No	3594 (86.7)
LTPA first trimester	
Yes	440 (10.6)
No	3707 (89.4)
LTPA second trimester	
Yes	363 (8.8)
No	3784 (91.2)
LTPA third trimester	
Yes	278 (6.7)
No	3869 (93.3)
Minutes of LTPA 1^st^ trimester	
0–149	3874 (93.4)
150– +	273 (6.6)
Minutes of LTPA 2^nd^ trimester	
0–149	3945 (95.1)
150– +	202 (4.9)
Minutes of LTPA 3^rd^ trimester	
0–149	4003 (96.5)
150– +	144 (3.5)
Physical activity tertiles (min.)	
First	146 (3.5)
Second	213 (5.1)
Third	194 (4.7)
Inactive women	3594 (86.7)

**Table 2:** Distribution of leisure-time physical activity in minutes (standard deviation) according to trimester of pregnancy and tertile of weekly activity, among women reporting any physical activity in the period.

	First trimester	Second trimester	Third trimester
First tertile	79.9 (38.4)	60.0 (31.8)	69.4 (39.2)
Second tertile	169.2 (78.2)	125.0 (46.8)	122.6 (59.8)
Third tertile	324.5 (140.7)	272.3 (110.9)	248.7 (114.7)
Time range of weekly physical activity among active women	20–840	10–630	15–630

Pelotas 2004 Birth Cohort (2004).

In Table 3 the development variables (Battelle's Inventory and IQ scores) are described according to LTPA information. Performances in the Battelle test for 12 and 24 months favor offspring from active women. At 48 months there was no clear pattern in Battelle's test to draw any conclusions. The crude analysis of IQ at 48 months showed that children from active women scored, on average, five points higher. All 8 PA-related variables studied presented significant results in favor of active women in crude analysis for IQ.

**Table 3:** Battelle's Development Inventory score (P90) and IQ scores (at 48 months) according to leisure-time physical activity (LTPA) information. Pelotas 2004 Birth Cohort (2004–2008).

	P90 of	p	P90 of	p	P90 of	p	IQ (mean)	p
	Battelle 12 m		Battelle 24 m		Battelle 48 m			
Any LTPA during pregnancy		<0.001		0.06		0.91		<0.001
Yes	15.1		10.9		6.2		103.9	
No	8.4		8.4		6.3		99.1	
Any LTPA 1^st^ trimester		0.001		0.06		0.39		<0.001
Yes	13.9		11.2		5.3		103.6	
No	8.7		8.4		6.4		99.3	
Any LTPA 2^nd^ trimester		<0.001		0.04		0.70		0.001
Yes	15.6		11.8		5.8		103.9	
No	8.7		8.4		6.3		99.4	
Any LTPA 3^rd^ trimester		0.001		0.18		0.79		0.0007
Yes	15.1		11.0		6.7		103.2	
No	8.9		8.6		6.3		99.5	
LTPA≥150 min/wk 1^st^ trimester		0.03		0.09		0.51		<0.001
Yes	13.1		11.6		5.3		104.6	
No	9.0		8.5		6.4		99.4	
LTPA≥150 min/wk 2^nd^ trimester		0.007		0.02		0.64		0.0001
Yes	14.9		13.5		5.5		103.8	
No	9.0		8.5		6.3		99.6	
LTPA≥150 min/wk 3^rd^ trimester		0.02		0.68		0.55		0.02
Yes	15.4		9.7		7.5		103.1	
No	9.1		8.7		6.3		99.7	
Physical activity tertiles (min.)		<0.001		0.12		0.82		<0.001
First	23.9		11.7		6.7		102.0	
Second	11.2		9.0		7.3		105.7	
Third	12.5		12.3		4.6		103.3	
Inactive women	8.4		8.4		6.3		99.1	

We have also analyzed the Battelle's score as a continuous variable (data not shown) and observed significant results in favor of active women in crude analysis. For the score measured at 12 (mean score  = 56.5 points) and 24 months (mean score  = 78.2 points), on average children from active women presented a significant advantage of 1 (one) point in all PA-related variables. At 48 months (mean score  = 37.0) most scores were still higher for active women, but none of the differences were significant.

After controlling for confounders (Table 4), only neurodevelopment at 12 months was associated with LTPA - children from women who were active during pregnancy [PR = 1.51 (95%CI: 1.17–1.94); p = 0.001] and active in first [PR = 1.33 (95%CI: 1.01–1.77); p = 0.04], second [PR = 1.50 (95%CI: 1.10–1.98); p = 0.009] and third [PR = 1.41 (95%CI: 1.01–1.97); p = 0.04] trimester presented higher scores at 12 months in the Battelle's test. And also, minutes of physical activity (in tertiles) were associated to being at or above the P90 at 12 months (p<0.001). At 24 months, all results were still higher for active women, but statistic significance was lost after adjustment. At 48 months, adjustment for confounders did not change the results for BDI, while differences in mean IQ were reduced to the point of not being significant any more.

**Table 4:** Multivariable analyses of the relationship between LTPA during pregnancy and neurodevelopmental variables.

	Battelle 12 m	p	Battelle 24 m	p	Battelle 48 m	p	IQ (β)	p
	PR (95%CI)		PR (95%CI)		PR (95%CI)			
Any LTPA during pregnancy		0.001		0.75		0.62		0.48
Yes	1.51 (1.17–1.94)		1.05 (0.79–1.39)		0.91 (0.63–1.32)		0.52	
No					1.00			
	1.00		1.00					
Any LTPA 1^st^ trimester		0.04		0.44		0.24		0.59
Yes	1.33 (1.01–1.77)		1.12 (0.84–1.51)		0.77 (0.50–1.19)		0.44	
No					1.00			
	1.00		1.00					
Any LTPA 2^nd^ trimester		0.009		0.550		0.44		0.73
Yes	1.48 (1.10–1.98)		1.10 (0.80–1.53)		0.83 (0.53–1.32)		−0.31	
No					1.00			
	1.00		1.00					
Any LTPA 3^rd^ trimester		0.04		0.89		0.85		0.78
Yes	1.41 (1.01–1.97)		1.03 (0.70–1.50)		1.05 (0.65–1.69)		−0.29	
No					1.00			
	1.00		1.00					
LTPA≥150 min/wk 1^st^ trimester		0.31		0.46		0.36		0.21
Yes	1.20 (0.84–1.71)		1.14 (0.80–1.63)		0.78 (0.45–1.34)		1.26	
No					1.00			
	1.00		1.00					
LTPA≥150 min/wk 2^nd^ trimester		0.07		0.21		0.50		0.90
Yes	1.42 (0.98–2.07)		1.28 (0.87–1.88)		0.81 (0.44–1.50)		−0.14	
No					1.00			
	1.00		1.00					
LTPA≥150 min/wk 3^rd^ trimester		0.14		0.86		0.60		0.72
Yes	1.40 (0.90–2.19)		0.95 (0.56–1.61)		1.18 (0.64–2.18)		−0.48	
No					1.00			
	1.00		1.00					
Physical activity tertiles (min.)		<0.001		0.86		0.88		0.76
First	2.53 (1.83–3.50)		1.19 (0.74–1.92)		0.99 (0.52–1.90)		−0.72	
Second	0.97 (0.62–1.53)		0.77 (0.47–1.27)		1.04 (0.61–1.76)		1.16	
Third	1.31 (0.86–1.98)		1.23 (0.82–1.84)		0.709 (0.35–1.39)		0.82	
Inactive women	1.00		1.00		1.00			

LTPA: leisure-time physical activity - PR: prevalence rates −95%CI: 95% confidence intervals.

Multivariable analyses included in the model: family income, schooling, smoking, skin color, maternal age, number of previous births, maternal occupational characteristics and preterm birth. Prevalence rates for the Battelle's outcomes derive from Poisson regression. Beta values for IQ scores derive from linear regression and indicate differences between inactive and active women.

Pelotas 2004 Birth Cohort (2004–2008).

In multivariable analysis of the continuous score (linear regression), we controlled for potential confounders (family income, schooling, smoking, skin color and preterm birth) and at 12 and 24 months all results favored active women but none was significant. At 48 months the scores for children from active women were slightly worst but, as in crude analysis, no significant results were observed.

## Discussion

We evaluated potential longitudinal effects of physical activity during pregnancy on children's development and IQ in the early years of infancy. This is the first study of its kind developed in a middle-income country and the birth cohort is being followed from 2004 to present time.

Our results agree with previous longitudinal studies [11], [12], [13], as we did not identify negative effects of physical activity during pregnancy on infant's development. Most crude results favor active women, but many were not statistically significant after controlling for potential confounders.

In a comparison between active and inactive women, Clapp et al. did not observe clinical significant between-group differences in performance on either the Bayley Scales of Infant Development or mental development scales, indicating that the offspring of exercising mothers have normal development in the first year of life [13]. Later, in a follow-up study, at 5 years of age, the motor, integrative, and academic readiness skills were similar between children from active and inactive women. However, children from exercising women performed significantly better on the Wechsler scales and tests of oral language skills [11]. Another study [12] indicated that neonatal behavior may be distinct as early as during the first week of life. Children from exercising women performed better in 2 of the 6 evaluations of the Brazelton Scale 5 days after birth. Performances were better in the ability to orient to environmental stimuli and ability to regulate their state or quiet themselves after sound and light stimuli. Meanwhile, the scores reflecting habituation, motor organization, autonomic stability and behavioral state range were not significantly different.

Physical activity in Brazil is highly associated to the sociodemographic characteristics (income and education), in general population and especially among pregnant women [6]. Development outcomes, such as IQ quotient, are known to be affected by maternal characteristics, mainly maternal education and by the home environment and stimulation [18], [19], [20], [21]. Children from active women presented higher IQ scores in all comparisons with inactive women, but maternal schooling and socioeconomic position may affect children's IQ and neurodevelopment [18], [19], [20], [21] and also are highly associated to physical activity, especially in Brazil [6]. In an alternative analysis, we observed that from the lower schooling category (0–4 years) to the highest category (12 years or more), the IQ score increases linearly 25% while a fivefold increase in mean minutes of physical activity during pregnancy was detected. Therefore, schooling is a potential confounder on the association between performance in development tests and maternal physical activity during pregnancy. Indeed, if schooling is not considered during multivariable analysis, all beneficial results are significant favoring active women.

Although our observational study cannot discuss causation, biologically, the potential effects of physical activity during pregnancy on neurodevelopment could possibly be explained by different pathways. First, glucose metabolism changes during pregnancy - gestational diabetes and maternal insulin resistance may change negatively intellectual and psychomotor development in children and result in attention disorders and lower motor coordination [22]. Previous studies have reported that intra uterine environment (maternal blood glucose) may affect children's cognitive abilities [23]. Physical activity's role in insulin control is recognized and could affect intrauterine glucose metabolism [22], [24]. Second, placental development is distinct between active and inactive women [25], [26] - physical activity results in better circulation and higher placental volumes, which could improve oxygen availability in uterus and influence neurologic development. Third, although aware of potential reverse causality, depression is frequently associated to physical inactivity [27] and inactive women are more likely to be depressed during pregnancy [28]. Gestational depression affects negatively psychological development in early infancy [29] and maternal well being and anxiety are associated to both physical activity and children's behavior [28], [30]. A sex-stratified analysis was carried out (data not shown) to assess potential gender differences and, although few results were changed, we noticed that all associations were stronger in magnitude among boys. Our results may be another indication that pregnancy characteristics perhaps affect distinctively girls' and boys' neurodevelopment during infancy [29].

It seems that the beneficial effects of physical activity, if real, weaken as the child ages, because the stronger (and significant) effects observed in our study were restrict to the first year of life.

Among the limitations of our study we must highlight the following issues: 1) the retrospective evaluation of physical activity could result in recall bias, however, our goal was to identify habitual activities to understand how usual behavior could influence the outcomes; 2) lack of intensity information was an option of the researchers because current intensity is already problematic to be assessed, thus past intensity was not a reliable information; 3) we also did not collect data on occupational, commuting or household activities. We only had information about standing or heavy lifting at worksite; however these variables were not associated with any of the outcomes, but were included in the multivariable model. On the other hand, few studies are available presenting longitudinal effects of LTPA during pregnancy, and our population sample also collaborates to the quality of our data with respect to potential selection bias. Loss to follow-up was no differential by physical activity status. From 2004 to 2008 we lost 8.4% of women, however the physical activity prevalence did not change significantly (13.3% vs. 13.5%).

Unfortunately, the amount of women reporting physical activity throughout the whole pregnancy or reporting physical activity during the third trimester is very small. Also, the percentage of women attaining the physical activity guidelines (150 minutes per week) in any of the trimesters was very little, and even with our large population sample, some of the associations tested could have been affected by lack of statistical power. For example, less than 4% of women achieved the recommended amount of PA in the third trimester.

As in any health study that considers physical activity as an exposure, we cannot rule out positive effects of different lifestyle characteristics that usually are associated to physical activity and were not evaluated. Physical activity is a voluntary behavior and it is plausible that, women who chose to exercise during gestation, also made other healthier choices in several aspects of pregnancy (food choices, for example) and during their children's early infancy. The maternal profile of women who are more health concerned may affect child's development in different manners and many characteristics cannot be considered in population studies or included in statistical analysis, resulting in potential residual negative confounding.

Based on our results, we conclude that LTPA during pregnancy does not seem to affect negatively children's neurodevelopment as children from active mothers presented better results for most of the studied outcomes. After controlling for confounders, children from women who were active in the first, second and third trimester of pregnancy presented significant better results at 12 months of age. Thus, PA should be advised to pregnant women based on all benefits already known [1], [10]. Child's improved neurodevelopment, especially in the first year of life, may be another positive effect of an active lifestyle during gestation.

## References

[1] Pivarnik JM, Chambliss HO, Clapp JF, Dugan SA, Hatch MC, et al (2006) Impact of physical activity during pregnancy and postpartum on chronic disease risk. Med Sci Sports Exerc 38: 989–1006. 16672855 10.1249/01.mss.0000218147.51025.8a

[2] Domingues MR, Barros AJ, Matijasevich A (2008) Leisure time physical activity during pregnancy and preterm birth in Brazil. Int J Gynaecol Obstet 103: 9–15. 18722614 10.1016/j.ijgo.2008.05.029

[3] Domingues MR, Matijasevich A, Barros AJ (2009) Physical activity and preterm birth: a literature review. Sports Med 39: 961–975. 19827862 10.2165/11317900-000000000-00000

[4] Devlieger R, Guelinckx I, Vansant G (2008) The impact of obesity on female reproductive function. Obes Rev 9: 181–182 author reply 183. 18257755 10.1111/j.1467-789X.2007.00460.x

[5] Guelinckx I, Devlieger R, Beckers K, Vansant G (2008) Maternal obesity: pregnancy complications, gestational weight gain and nutrition. Obes Rev 9: 140–150. 18221480 10.1111/j.1467-789X.2007.00464.x

[6] Domingues MR, Barros AJ (2007) Leisure-time physical activity during pregnancy in the 2004 Pelotas Birth Cohort Study. Rev Saude Publica 41: 173–180. 17420903 10.1590/s0034-89102007000200002

[7] Haakstad LA, Voldner N, Henriksen T, Bo K (2009) Why do pregnant women stop exercising in the third trimester? Acta Obstet Gynecol Scand 88: 1267–1275. 19824869 10.3109/00016340903284901

[8] Duncombe D, Wertheim EH, Skouteris H, Paxton SJ, Kelly L (2009) Factors related to exercise over the course of pregnancy including women's beliefs about the safety of exercise during pregnancy. Midwifery 25: 430–438. 18063253 10.1016/j.midw.2007.03.002

[9] Committee on Obstetric Practice (2002) ACOG committee opinion. Exercise during pregnancy and the postpartum period. Number 267, January 2002. American College of Obstetricians and Gynecologists. Int J Gynaecol Obstet 77: 79–81. 12053898 10.1016/s0020-7292(02)80004-2

[10] Haskell WL, Lee IM, Pate RR, Powell KE, Blair SN, et al (2007) Physical activity and public health: updated recommendation for adults from the American College of Sports Medicine and the American Heart Association. Circulation 116: 1081–1093. 17671237 10.1161/CIRCULATIONAHA.107.185649

[11] Clapp JF 3rd (1996) Morphometric and neurodevelopmental outcome at age five years of the offspring of women who continued to exercise regularly throughout pregnancy. J Pediatr 129: 856–863. 8969727 10.1016/s0022-3476(96)70029-x

[12] Clapp JF 3rd, Lopez B, Harcar-Sevcik R (1999) Neonatal behavioral profile of the offspring of women who continued to exercise regularly throughout pregnancy. Am J Obstet Gynecol 180: 91–94. 9914584 10.1016/s0002-9378(99)70155-9

[13] Clapp JF 3rd, Simonian S, Lopez B, Appleby-Wineberg S, Harcar-Sevcik R (1998) The one-year morphometric and neurodevelopmental outcome of the offspring of women who continued to exercise regularly throughout pregnancy. Am J Obstet Gynecol 178: 594–599. 9539531 10.1016/s0002-9378(98)70444-2

[14] Barros AJ, Santos Ina S, Victora CG, Albernaz EP, Domingues MR, et al (2006) [The 2004 Pelotas birth cohort: methods and description.]. Rev Saude Publica 40: 402–413. 16810363 10.1590/s0034-89102006000300007

[15] Santos IS, Barros AJ, Matijasevich A, Domingues MR, Barros FC, et al.. (2010) Cohort Profile: The 2004 Pelotas (Brazil) Birth Cohort Study. Int J Epidemiol. 10.1093/ije/dyq130 PMC3235016 20702597

[16] Newborg J, Stock J, Wnek L, Guidabaldi J, Svinicki J (1988) Battelle Developmental Inventory. Itasca, IL: Riverside Publishing.

[17] Wechsler D (1967) Wechsler Preschool and Primary Scale of Intelligence. New York: Psychological Corporation; 1967.

[18] Huisman M, Araya R, Lawlor DA, Ormel J, Verhulst FC, et al. Cognitive ability, parental socioeconomic position and internalising and externalising problems in adolescence: findings from two European cohort studies. Eur J Epidemiol 25: 569–580. 20535529 10.1007/s10654-010-9473-1 PMC2921071

[19] Santos DN, Assis AM, Bastos AC, Santos LM, Santos CA, et al (2008) Determinants of cognitive function in childhood: a cohort study in a middle income context. BMC Public Health 8: 202. 18534035 10.1186/1471-2458-8-202 PMC2442073

[20] To T, Guttmann A, Dick PT, Rosenfield JD, Parkin PC, et al (2004) Risk markers for poor developmental attainment in young children: results from a longitudinal national survey. Arch Pediatr Adolesc Med 158: 643–649. 15237063 10.1001/archpedi.158.7.643

[21] Barros AJ, Matijasevich A, Santos IS, Halpern R (2010) Child development in a birth cohort: effect of child stimulation is stronger in less educated mothers. Int J Epidemiol 39: 285–294. 19717543 10.1093/ije/dyp272 PMC2817089

[22] Ornoy A, Ratzon N, Greenbaum C, Wolf A, Dulitzky M (2001) School-age children born to diabetic mothers and to mothers with gestational diabetes exhibit a high rate of inattention and fine and gross motor impairment. J Pediatr Endocrinol Metab 14 Suppl 1681–689. 11393563 10.1515/jpem.2001.14.s1.681

[23] Fraser A, Nelson SM, Macdonald-Wallis C, Lawlor DA (2012) Associations of Existing Diabetes, Gestational Diabetes, and Glycosuria with Offspring IQ and Educational Attainment: The Avon Longitudinal Study of Parents and Children. Exp Diabetes Res 2012: 963735. 22927834 10.1155/2012/963735 PMC3425081

[24] Silverman BL, Rizzo TA, Cho NH, Metzger BE (1998) Long-term effects of the intrauterine environment. The Northwestern University Diabetes in Pregnancy Center. Diabetes Care 21 Suppl 2B142–149. 9704242

[25] Clapp JF (2006) Influence of endurance exercise and diet on human placental development and fetal growth. Placenta 27: 527–534. 16165206 10.1016/j.placenta.2005.07.010

[26] Clapp JF 3rd, Kim H, Burciu B, Lopez B (2000) Beginning regular exercise in early pregnancy: effect on fetoplacental growth. Am J Obstet Gynecol 183: 1484–1488. 11120515 10.1067/mob.2000.107096

[27] Strohle A (2009) Physical activity, exercise, depression and anxiety disorders. J Neural Transm 116: 777–784. 18726137 10.1007/s00702-008-0092-x

[28] Poudevigne MS, O′Connor PJ (2006) A review of physical activity patterns in pregnant women and their relationship to psychological health. Sports Med 36: 19–38. 16445309 10.2165/00007256-200636010-00003

[29] Gerardin P, Wendland J, Bodeau N, Galin A, Bialobos S, et al. Depression during pregnancy: is the developmental impact earlier in boys? A prospective case-control study. J Clin Psychiatry 72: 378–387. 21208585 10.4088/JCP.09m05724blu

[30] Hernandez-Martinez C, Arija V, Balaguer A, Cavalle P, Canals J (2008) Do the emotional states of pregnant women affect neonatal behaviour? Early Hum Dev 84: 745–750. 18571345 10.1016/j.earlhumdev.2008.05.002

